# Complete Genome Sequence of a Variant Pseudorabies Virus Strain Isolated in Central China

**DOI:** 10.1128/genomeA.00149-16

**Published:** 2016-03-17

**Authors:** Shuangshuang Xiang, Zhi Zhou, Xule Hu, Yingying Li, Chaolin Zhang, Juan Wang, Xiangdong Li, Feifei Tan, Kegong Tian

**Affiliations:** a College of Animal Science and Veterinary Medicine, Henan Agricultural University, Zhengzhou, People’s Republic of China; b National Research Center for Veterinary Medicine, High-Tech District, Luoyang, People’s Republic of China; c Veterinary Diagnosis Center and OIE Porcine Reproductive and Respiratory Syndrome Laboratory, China Animal Disease Control Center, Chaoyang District, Beijing, People’s Republic of China

## Abstract

Pseudorabies virus (PRV) variants have been prevalent in China since 2011 and have caused huge economic losses to the Chinese pig industry. Here, we report the genome sequence of a PRV variant HN1201 that was isolated from diseased animals in central China in 2011.

## GENOME ANNOUNCEMENT

Pseudorabies virus (PRV) is a double strand DNA virus of the genus *Varicellovirus*, subfamily *Alphahepersvirinae*, and family *Herpesviridae*, which causes pseudorabies (PR), or Aujeszkys’ disease, in livestock and wild animals (1). PR has been effectively controlled by using attenuated live or inactivated vaccines in China since the 1970s. However, highly pathogenic PRV variants with clinical manifestations of high fever, depression, anorexia, cough, shivering, diarrhea, and systemic neurological symptoms with high mortality started to emerge and became prevalent in Bartha-K61 vaccinated pig herds in late 2011 (2, 3). Since then, the virus has spread nationwide and caused huge economic losses to the Chinese pig industry.

In this study, one PRV variant, designated HN1201, was successful isolated from brain tissue specimens of diseased pigs in Henan province (3). The virus was seeded in porcine kidney (PK-15) cells for virus isolation. Virus DNA was extracted from the 2nd passage of the virus and subjected to sequencing using Illumina MiSeq technology as described previously (4). The genome was analyzed and spliced using DNAStar Lasergene v7.1 (DNASTAR Inc, Madison, WI, USA). Here, we report the complete genome sequence of PRV variant HN1201 with unique genetic changes in different proteins.

The complete genome of PRV strain HN1201 is 144,174 bp in length with 73.54% G+C content. HN1201 shares 95.6%, 95.9%, and 97.5% genome similarity with PRV Kaplan (access number KJ717942), Becker (JF797219), and Bartha (JF797217), respectively. The long unique (U_L_) and short unique (Us) regions are 94,200 bp and 12,168 bp in size, respectively, and the terminal repeats (TRs) and inverted repeats (IRs) flanking Us are 11,117 bp in size. The HN1201 genome includes 69 open reading frames (ORFs), and some ORFs show extensive variations compared with previously isolated PRV strains.

The most variant genetic changes of HN1201 are located in the glycoproteins, including glycoprotein B, C, E, and I, which play important roles in virus entry, egress, cell-to-cell spread, and modulation of immune responses (5). HN1201 glycoprotein B (UL27) contains a continuous 3-amino acid deletion (SPG) at positions 75 to 77 and a 1-amino acid insertion at position 94 (G). Glycoprotein C (UL44) has a 7-amino acid insertion at 63 to 69 (AAASTPA) and six interspersed substitutions compared with previous isolates. HN1201 glycoprotein E (US8) contains a 2-amino acid insertion at positions 48 (D) and 496 (D) and glycoprotein I contains a 1-amino acid deletion at position 172 (H) and a 1-amino acid insertion at position 238 (G). To our knowledge, this is the first reported genome sequence of PRV variant isolated in China. The analysis of variations in HN1201 viral proteins may enable us to figure out the molecular epidemiology of PRV and will be helpful for making effective control strategies to reduce the loss of swine production in China.

### Nucleotide sequence accession number.

The PRV strain HN1201 genome sequence has been deposited in GenBank with the accession number KP722022.
